# Characteristics associated with willingness to participate in a randomized controlled behavioral clinical trial using home-based personal computers and a webcam

**DOI:** 10.1186/1745-6215-15-508

**Published:** 2014-12-23

**Authors:** Hiroko H Dodge, Yuriko Katsumata, Jian Zhu, Nora Mattek, Molly Bowman, Mattie Gregor, Katherine Wild, Jeffrey A Kaye

**Affiliations:** Layton Aging and Alzheimer’s Disease Center, Department of Neurology, Oregon Health & Science University, Portland, OR USA; Michigan Alzheimer’s Disease Center, Department of Neurology, University of Michigan, Ann Arbor, MI USA; Oregon Center for Aging and Technology, Oregon Health & Science University, Portland, OR USA; College of Public Health, University of Kentucky, Lexington, KY USA; Graduate School of Public Health, University of Michigan, Ann Arbor, MI USA; Portland Veteran Affairs Medical Center, Portland, OR USA

**Keywords:** Sample recruitment selection bias, Volunteer bias, Behavioral randomized controlled trial, PC, Internet, Webcam, Conversation-based social interaction, Cognitive function, Mild cognitive impairment

## Abstract

**Background:**

Trials aimed at preventing cognitive decline through cognitive stimulation among those with normal cognition or mild cognitive impairment are of significant importance in delaying the onset of dementia and reducing dementia prevalence. One challenge in these prevention trials is sample recruitment bias. Those willing to volunteer for these trials could be socially active, in relatively good health, and have high educational levels and cognitive function. These participants’ characteristics could reduce the generalizability of study results and, more importantly, mask trial effects. We developed a randomized controlled trial to examine whether conversation-based cognitive stimulation delivered through personal computers, a webcam and the internet would have a positive effect on cognitive function among older adults with normal cognition or mild cognitive impairment. To examine the selectivity of samples, we conducted a mass mail-in survey distribution among community-dwelling older adults, assessing factors associated with a willingness to participate in the trial.

**Methods:**

Two thousand mail-in surveys were distributed to retirement communities in order to collect data on demographics, the nature and frequency of social activities, personal computer use and additional health-related variables, and interest in the prevention study. We also asked for their contact information if they were interested in being contacted as potential participants in the trial.

**Results:**

Of 1,102 surveys returned (55.1% response rate), 983 surveys had complete data for all the variables of interest. Among them, 309 showed interest in the study and provided their contact information (operationally defined as the committed with interest group), 74 provided contact information without interest in the study (committed without interest group), 66 showed interest, but provided no contact information (interest only group), and 534 showed no interest and provided no contact information (no interest group). Compared with the no interest group, the committed with interest group were more likely to be personal computer users (odds ratio (OR) = 2.78), physically active (OR = 1.03) and had higher levels of loneliness (OR = 1.16).

**Conclusion:**

Increasing potential participants’ familiarity with a personal computer and the internet before trial recruitment could increase participation rates and improve the generalizability of future studies of this type.

**Trial registration:**

The trial was registered on 29 March 2012 at ClinicalTirals.gov (ID number NCT01571427).

## Background

Faced with an aging population and a growing number of people with dementia, it is critical that we develop effective pharmacological and non-pharmacological prevention treatments. The majority of pharmacological phase III randomized controlled trials have failed thus far due to lack of efficacy and/or toxicity. Thus, developing behavioral intervention strategies to delay or prevent cognitive decline has increasing importance for public health benefits. As shown by a recent study [1], the population effect size of behavioral modifications could be high. One important issue that is especially critical in behavioral intervention trials is sample recruitment selection bias. That is, those who are willing to participate in behavioral interventions or remain in the intervention trial might differ in various characteristics from those who are not willing to participate or drop out. Sample selection bias can also occur in pharmacological trials, but most behavioral intervention trials require changes in lifestyle at least to some degree, and therefore selection bias could potentially be magnified on this basis in contrast to pharmacological trials.

Epidemiological studies have demonstrated that larger social networks, or more frequent social interactions, could have potential protective effects on the incidence of dementia (for example, see review by Fratiglioni and colleagues [2]). To determine elements of social networks that may play a key role in mitigating cognitive decline and its efficacy, and ultimately to turn this knowledge into actionable programs, we examined whether face-to-face conversation - a core component of social interaction - can enhance cognitive function through stimulating social cognition. To facilitate efficiency and quantification of outcomes, we utilized information communication technologies including personal computers (PCs), webcams and the internet. One issue that we tried to identify before the trial started was characteristics of a potential participant pool from which we recruited our study participants. Volunteers who participate in research studies are generally high-functioning individuals with active lifestyles and higher educational attainment [3, 4]. This can reduce the generalizability of trial results (that is, volunteer bias). This concern might be particularly relevant for the above trial which encourages social interaction using contemporary communication technologies. Accordingly, we examined factors associated with willingness to participate in the trial by distributing surveys in the community. The survey was also used as a part of our recruitment strategy in the above mentioned trial [5].

## Methods

### Mail-in survey

We distributed 2,000 survey questionnaires targeting those living in retirement communities and senior centers located in the Portland metropolitan area, approximately within a 1 hour commute from Oregon Health & Science University (OHSU). Sixteen communities and centers that cover a wide range of socioeconomic status (including low income household retirement communities designated by the municipal government) and that had agreed to collaborate for research studies with OHSU were included. To increase response rates, we conducted information sessions at each community and center explaining the upcoming trials. The survey was distributed at the conclusion of the information session and also distributed by mail through the retirement communities and senior center administrative offices. The survey was returned to our study office located at OHSU by using a pre-addressed/stamped envelope. In addition to this community-specific recruitment, we also sent a small number of mail-in survey questionnaires (n = 126) using the Layton Aging & Alzheimer’s Disease Center, also known as the Oregon Alzheimer’s Disease Center (OADC), volunteer list where names, telephone numbers and addresses of those interested in participating in the studies offered by OADC are retained. The majority of this list consists of participants who signed up to be enrolled as normal controls in past studies.

The information collected in the survey includes: demographic information (age, gender, years of education, living arrangement, marital status, number of children), nature and frequencies of social/cognitive/physical activities, self-rated health, loneliness measurement, activities of daily living and instrumental activities of daily living, and brief questions on internet and PC usage. Additionally, we asked willingness (yes/no) to participate in the future clinical trial (after brief explanations of prevention study protocol) and provide contact information if they wish to be contacted - that is, we asked the survey respondent: (1) whether he or she is interested in participating in the trial (yes/no), and (2) to provide their contact information (name, address and phone number) indicating that they would like to be contacted as potential participants in the trial. Subjects were informed that they could decline to participate any time after hearing about the study. These variables are explained in detail below. In the current study, characteristics of the survey respondents associated with having an interest in participating in the proposed trial and/or providing contact information were assessed. The research protocol was approved by the Institutional Review Board of OHSU, Portland, Oregon, USA. The full trial design and the main results of the above intervention trial are described in detail elsewhere [5] and all subjects who enrolled into the trial signed written informed consent (IRB# 00005590).

### Operational criteria used to define levels of interest in participating in the study

In the survey, we used the following phrase to introduce the upcoming trial: “In the near future, we are going to conduct a study where we will talk with seniors daily for about 30 minutes using the Internet and a computer video camera. The purpose of this study will be to see how communications and frequent social interactions affect our thinking abilities”. Then we asked respondents: (A) “Does this type of study interest you? We will not contact you unless you indicate that you want to be contacted below”, (B) “Would you like to be contacted by our study coordinator as a potential participant in a future study as above?” And if yes, then asked (C) “Could you provide your contact information?” (name, telephone number, and address (address is optional)). Four outcomes were created operationally using the combination of the answers to the above questions: (1) showed interest in the type of study (that is, selected “yes” to question A), and also provided their contact information (henceforth called “committed with interest"); (2) did not show interest in the study itself (that is, selected “no” to question A), but provided contact information in any case (that is, not interested in the study topic but, if needed, does not mind helping the study by participating, henceforth called “committed without interest”); (3) showed interest but did not provide contact information (henceforth called “interest without commitment”); and (4) did not show interest nor provide contact information (henceforth called “no interest”). We were particularly interested in examining whether those “committed with interest" (potential participants in the subsequent trial) differed in regard to their levels of social engagement (social/cognitive and physical activities), loneliness, and PC usage as compared to the “no interest” group.

### Variables included in the model

#### Physical and social/cognitive activity indices

We created summary activity scores for physical and social/cognitive activities using a method similar to that described in Dodge and colleagues [6]. Briefly, we asked about various activities and their average frequencies of engagement. Frequencies were assessed as: 1, never; 2, less than once a year; 3, a few times a year; 4, a few times a month; 5, a few times a week; and 6, almost every day. The activity index was created by summing each frequency, with higher scores showing higher engagement. Examples of social/cognitive activities include reading a newspaper, reading a book, playing games such as cards and chess, hobbies such as knitting, paper crafts, drawing, woodworking, and so forth. Examples of physical activities include yoga, stretching, walking, jogging, dancing, swimming, and so forth. A full list of assessed activities is available upon request to the corresponding author.

#### The loneliness scale

We used a measurement developed by Hughes and colleagues [7] that consists of three questions: (1) how often do you feel that you lack companionship?; (2) how often do you feel left out?; and (3) how often do you feel isolated from others? Composite scores indicating loneliness were created by summing the frequency scores (1 = hardly ever, 2 = some of the time, 3 = often). Higher scores indicate higher levels of loneliness.

#### Personal computer usage

PC usage was ascertained by asking: “do you use a personal computer? Yes/No”. If yes, participants were asked “how often do you use it: 1, less than once a year; 2, a few times a year; 3. a few times a month; 4, a few times a week; and 5, almost every day. First we included PC usage as a categorical variable (yes/no) in the statistical model, and as a secondary analysis we also included the frequency of usage.

#### Self-rated health

Self-rated health was included as a general health indicator (poor versus fair, good, or excellent). The control variables included in the model are: age (<65, 65–74, 75–79, 80–84, 85–89, and 90 years and older), gender, marital status, and years of education.

### Statistical methods

Characteristics associated with each of the four groups (“no interest”, “interest without commitment”, “committed with interest”, “committed without interest”) were first analyzed with univariate chi-square statistics (categorical variables) and *t* test or Wilcoxon ranked sum tests (continuous variables). Multinomial logit model was run with the no interest group as a reference group and the other three groups as outcomes. SAS 9.2 (Cary, NC, USA) was used for analyses.

## Results

Out of 2,000 surveys distributed, 1,102 surveys were returned (55.1% response rate). Among them, 119 (10.8%) were missing at least one of the variables of our interest examined here and thus were excluded from further analysis. Thus, 983 subjects were used in the subsequent analysis. Compared with those included in the analysis, those excluded from the analysis were more likely to have lower educational attainment (*P* = 0.0002, Wilcoxon ranked sum test), but were not different in age (*P* = 0.11) or gender (*P* = 0.26). As seen in Table 1, 309 (31.4%) were categorized as “committed with interest”, 74 (7.5%) as “committed without interest”, 66 (6.7%) as “interested without commitment”, and 534 (54.3%) as “no interest”. PC usage, physical and cognitive activity indices, and age distributions differed significantly between groups (*P* < 0.0001). Results of the multinomial logit model are presented in Table 2. Compared with the no interest group, the committed with interest group were younger (odds ratio (OR) = 0.83, *P* = 0.001), more likely to be PC users (OR = 2.78, *P* < 0.0001), be physically active (OR = 1.03, *P* = 0.01), and had a higher loneliness score (OR = 1.16, *P* = 0.02). The committed without interest group were younger (OR = 0.79, *P* = 0.01), and more physically active (OR = 1.06, *P* = 0.005). The interest only group more likely to be PC users (OR = 2.57, *P* = 0.02). As a secondary analysis, we also examined frequency of PC use. The results remained unchanged.

**Table 1:** **Baseline characteristics of survey respondents by level of interest**

Covariates (range)	No interest group n = 534	Interest without commitment group n = 66	Commitment without interest group n = 74	Commitment with interest group n = 309	Difference among groups ( ***P***-values) ^*^
Age group					<0.0001
62–74 (%)	15.1%	23.1%	23.1%	29.3%	
75–84 (%)	37.8%	38.5%	50.0%	36.7%	
85 and above (%)	47.2%	38.5%	26.9%	34.0%	
Female (%)	72.0%	66.2%	74.0%	69.8%	0.63
Married (%)	61.9%	62.8%	59.0%	55.1%	0.21
Years of education (2–32)	15.5 (3.0)	16.4 (3.3)	16.1 (3.1)	15.8 (2.9)	0.03
Social activity index (0–27)^a^	17.0 (5.7)	17.7 (5.7)	17.8 (5.5)	17.4 (5.5)	0.42
Cognitive activity index (0–45)^a^	23.7 (8.9)	25.2 (10.0)	25.5 (8.9)	26.0 (7.9)	0.001
Physical activity index (0–64)^a^	9.3 (6.4)	9.5 (6.2)	12.3 (7.7)	11.2 (7.1)	<0.0001
Loneliness scale (3-9)^b^	3.9 (1.3)	4.0 (1.4)	4.0 (1.4)	4.1 (1.6)	0.14
Self-rated health (% poor versus others)	2.7%	1.3%	2.6%	4.2%	0.45
Personal computer usage^c^ (% yes)	63.0%	82.4%	74.7%	85.1%	<0.0001

^*^Pearson chi-square test for categorical variables and *t* test or Wilcoxon ranked sum test for continuous variables.

^a^Composite scores were created by summing the self-reported frequencies: 1, never; 2, less than once a year; 3, once or twice a year; 4, several times a year; 5, about once a month; 6, every week; 7, several times a week; for various cognitive, social and physical activities.

^b^Three-item scale developed by Hughes and colleagues [7]. Questions were phrased as: 1, how often do you feel that you lack companionship?; 2, how often do you feel left out?; 3, how often do you feel isolated from others? Composite scores were calculated by taking the sum of frequency scores: 1, hardly ever; 2, some of the time; and 3, often.

^c^“Do you use a personal computer?” (Yes/No).

**Table 2:** **Multinomial logit model results: characteristics associated with level of interest in trial participation (n = 983; as compared to the “no interest” reference group)**

	Committed with interest			Committed without interest			Interest only		
Covariates	OR	95% CI	***P***-value	OR	95% CI	***P***-value	OR	95% CI	***P***-value
Age group (1-6)^a^	0.83	(0.74, 0.92)	0.001^**^	0.79	(0.65, 0.95)	0.01^**^	0.91	(0.74, 1.10)	0.33
Female (vs. male)	0.89	(0.63, 1.25)	0.49	1.10	(0.60, 2.00)	0.76	0.75	(0.42, 1.36)	0.35
Married (vs. unmarried)	0.81	(0.60, 1.11)	0.19	0.88	(0.52, 1.50)	0.64	0.90	(0.51, 1.58)	0.71
Years of education	1.00	(0.94, 1.05)	0.91	1.10	(0.99, 1.21)	0.07	1.08	(0.98, 1.19)	0.11
Social activity index^b^	0.98	(0.95, 1.02)	0.33	1.01	(0.96, 1.07)	0.62	1.01	(0.95, 1.06)	0.85
Cognitive activity index^b^	1.01	(0.99, 1.04)	0.20	1.00	(0.96, 1.03)	0.84	1.02	(0.98, 1.06)	0.24
Physical activity index^b^	1.03	(1.01, 1.06)	0.01^**^	1.06	(1.02, 1.10)	0.005^**^	0.98	(0.94, 1.03)	0.43
Loneliness scale^c^	1.16	(1.02, 1.28)	0.02^*^	1.08	(0.89, 1.32)	0.41	1.18	(0.97, 1.43)	0.10
Self-rated health (poor vs. others)	1.71	(0.72, 4.05)	0.23	1.27	(0.26, 6.14)	0.77	0.73	(0.09, 5.93)	0.77
Personal computer usage^d^	2.78	(1.80, 4.02)	<0.0001^**^	1.19	(0.62, 2.30)	0.60	2.57	(1.16, 5.60)	0.02^*^

^**^Significant at *P* < 0.05; ^**^significant at *P* < 0.01.

^a^Ordered age groups as used in the survey questionnaire: 1, 62 years or younger (a reference group); 2, 63–74 years; 3, 75–79 years; 4, 80–84 years; 5, 85–89 years; 6, 90 years and older.

^b^Composite scores were created by summing the self-reported frequencies: 1, never; 2, less than once a year; 3, once or twice a year; 4, several times a year; 5, about once a month; 6, every week; 7, several times a week; for various cognitive, social and physical activities.

^c^Three-item scale developed by Hughes and colleagues [7]. Questions were phrased as: 1, how often do you feel that you lack companionship?; 2, how often do you feel left out?; 3, how often do you feel isolated from others? Composite scores were calculated by taking the sum of frequency scores: 1, hardly ever; 2, some of the time; and 3, often.

^d^“Do you use a personal computer?” (Yes/No).

CI, Confidence interval; OR, odds ratio.

Finally as a *post-hoc* analysis, we examined the reasons for not being interested in participating in the study. Out of 534 subjects in the “no interest” group, 524 subjects selected one or more reasons provided in the survey or wrote a reason as an open-ended response. The response categories provided in the survey and frequencies are: 1) I am too busy to participate (n = 161, 30.1% of the “no interest” group); 2) I do not like using technologies such as webcam and internet (n = 158, 29.5%); 3) I do not participate in any studies (n = 99, 18.5%); and (4) I do not like to talk with someone daily (n = 81, 16.1%). Additionally 11 respondents (2%) reported an open-ended response that they have not used a PC, the internet or a webcam, and 16 respondents (3%) wrote simply “not interested”.

## Discussion

Developing behavioral prevention studies for cognitive decline is of significant importance to those at risk for dementia. However, those who volunteer in behavioral prevention studies could differ in various characteristics from those who do not volunteer. This selection bias could limit the interpretation of trial results. We developed a randomized controlled trial which aimed to improve cognitive function using contemporary technologies such as a PC, webcam and the internet. As a part of our recruitment strategy and to better understand the characteristics of the sampling pool from which we drew our trial participants, we distributed 2,000 mail-in surveys and asked whether respondents would be interested in participating in our study.

Our mail-in survey study results showed that current PC users had more than two-fold higher odds of showing interest in a prevention study that uses information communication technologies than those who were not current PC users. Those who provided contact information (committed with interest group; the group we used as a recruitment sampling pool for our prevention study) were more likely to be younger, current PC users, physically active, and have higher loneliness levels, compared with the no interest group (those who did not show interest and provided no contact information). This suggests that when we analyze the results of the above clinical trial, we should examine whether different efficacy is seen when comparing PC users and non-users so that we can estimate our potential trial efficacies among non-participants who are more likely to be non-PC users. Two dominant reasons reported for not being interested in study participation were being too busy and not liking to use these technologies. According to a US census bureau report, in 2010, 55% of adults 65 years and older were living in a household with internet access, and 42% of these individuals accessed internet at home [8]. It can be anticipated following the ongoing trends in information communication technology use among the older population that the prevalence of internet and PC users will further increase as baby boomers move into the retirement age group. Nevertheless, there is still high anxiety and lack of confidence about computer usage among the current generation of older adults [9]. In our randomized controlled trial, we provided PCs, internet services and monitors to the participants (that is, participants did not need to have these items). For daily conversational sessions, we created a user-friendly system such that participants did not need to know how to use a computer, other than to touch a touch screen preconfigured to receive calls and automatically begin the conversational session. Clarification in the survey description that previous PC and internet experience was not required for participation in our study could have increased the number of those who showed interest.

Volunteer bias, when a particular sample contains only those participants who are actually willing to participate in the study or experiment, is well documented [10–13]. Individuals who volunteer for research studies are often more highly educated, have higher socio-economic status and are more sociable. Volunteer bias has been reported in an exercise intervention trial where participants were found to be fitter and healthier than non-volunteers [11]. Other studies have also suggested that volunteer bias might affect clinical trial results [12, 13]. One recent study showed that adjusting volunteer bias using propensity scores modified the study results [14]. In this study, structural magnetic resonance imaging brain scans were assessed among volunteers in an existing population-based cohort study. They found that, compared with those not interested, the potentially interested individuals were significantly younger, more likely to be male, better educated, generally healthier, and more likely to be cognitively intact and dementia-free. Furthermore, weighted models adjusted for selection bias using propensity scores showed fewer regions of interest to be associated with mild cognitive impairment than were in the original unweighted models. This study was unique in that magnetic resonance imaging assessment was nested within a well-characterized longitudinal cohort and thus sample selection bias (volunteer bias) and changes in results through adjusting for the bias could be assessed. Most clinical trials with cognitive function outcomes try not to recruit participants from existing cohorts in order to avoid the bias coming from multiple exposures to neuropsychological tests (for example, practice effects [15]), and therefore measuring volunteer bias accurately and its potential effect on the results are challenging. A recent systematic review and meta-analysis study by Cooper and colleagues [16] showed that few randomized controlled behavioral trials have investigated how to increase recruitment to intervention studies. Bower and colleagues [17] summarized various methods used to encourage recruitment and retention in intervention studies by using reported survey data from Clinical Trials Units in the United Kingdom. They concluded that recruitment remains a significant challenge and further adoption of innovative methods to develop, test, and implement recruitment interventions are required [17]. Trial efficacy and external validity depend on who are selected into the study. Identifying characteristics of those less likely to participate into given trials before the actual trial starts is encouraged in any trials so that strategized recruitment efforts can be delivered to improve the generalizability of the trial results, as shown in some studies [18].

There were some limitations of this mail-in survey study. Although our overall response rate of 55% is relatively high for mail-in surveys, we only know the characteristics of those who responded to our survey. There could be additional unknown selection biases between those who responded to our survey versus those who did not. We distributed surveys to those living in retirement communities or in contact with community centers. Although over 60% of non-institutionalized adults aged 80 years and older were living in retirement communities in 2010 in the counties where we distributed the survey (unpublished data, obtained directly from the local Multnomah county office of Area Agency on Aging), those living in retirement communities could be different from those living in free-standing single family homes in many aspects and this may limit the generalizability of our study results. Survey distribution was limited to the Portland metropolitan area approximately within a 1 hour driving distance from our study site. We expect that older adults in other areas (for example, those living in different states or in rural locations) may have different response patterns associated with different levels of exposure to information technology.

## Conclusions

Through our analysis of 983 surveys completed by older adults we found that those who were in the “committed with interest” group were more likely to be PC users, physically active, and reported higher loneliness scores, with being PC users in particular showing a strong association. Increasing growth in adoption and familiarity with contemporary information and communication technologies in the aging community should improve the generalizability of future trials of this type to a larger proportion of the aging population.

## Abbreviations

OADC: Oregon Alzheimer’s Disease Center
OHSU: Oregon Health & Science University
OR: odds ratio
PC: personal computer.
